# A consideration of the potential benefits and harms of menopausal hormone treatment

**DOI:** 10.1371/journal.pmed.1004567

**Published:** 2025-04-10

**Authors:** Ming Jun Kuck, Eef Hogervorst

**Affiliations:** National Centre for Exercise Medicine, Loughborough University, Loughborough, England

## Abstract

Ming Jun Kuck and Eef Hogervorst consider the data on the safety and potential cognitive effects in later life of midlife menopausal hormone treatment in women.

Women have a 1.56 times higher risk of dementia in many cohorts, and this was hypothesized to be partly due to the loss of estrogens after menopause. There is evidence from preclinical studies, as well as observational data and user experiences, which support the possibility that estrogens could protect the aging brain [1]. However, the debate over the potential benefits of menopausal hormone therapy (MHT, which includes estrogens) to prevent cognitive decline and lower dementia risk in women has been ongoing for more than seven decades. For instance, recent European Society of Human Reproduction and Embryology (ESHRE) guidelines suggest that there is “strong evidence” to support the benefits of MHT on brain health for women undergoing early and premature menopause [2]. However, updated National Institute for Health and Care Excellence (NICE) guidelines concluded that there is insufficient consistent and good-quality evidence for early (or later) menopausal women from randomized controlled trials (RCTs) or observational studies to establish the benefits of MHT in maintaining brain function. In fact, the studies reviewed by NICE reported possible increased dementia risk with MHT, when started over the age of 65 [3]. The question remains whether MHT in midlife can affect the risk of poor cognition and dementia in later life.

Gleason and her team conducting the Kronos Early Estrogen Prevention Study (KEEPS)-Continuation study have provided another important piece to the puzzle of the long-term effects of midlife MHT exposure on later life cognition [4]. During the original KEEPS-Cog trial, data were collected from 299 women who had been given MHT (either transdermal 17β-estradiol or oral conjugated equine estrogens [oCEE], both with micronized progesterone) or placebo close to their age at menopause for 48 months [5]. Two hundred seventy-five women from the original group of 299 participated in the KEEPS continuation study 10 years later [4], when their cognitive performance was assessed using the same instruments. The authors evaluated whether previous exposure to MHT affected later-life cognition while accounting for age, education, and *APOE*e*4* carrier status using linear latent growth models. The results indicated no long-term cognitive effects of exposure to MHT when started close to the age at menopause versus placebo. Sensitivity analyses excluding women who had reported continued MHT after the trial ended (14% of the total group) also did not show different outcomes. The follow-up findings suggested long-term neurocognitive safety of MHT when initiated shortly after menopause in healthy women.

However, participants of KEEPS were mainly non-Hispanic white individuals who were well-educated, free from comorbid conditions, and with low cardiovascular disease (CVD) risk at baseline, which limits the generalization of results. In contrast, previous RCT, such as the renowned Women’s Health Initiative Study (WHI), which recruited over 16,608 women from diverse ethnic backgrounds, along with its ancillary study, the Women’s Health Initiative Memory Study (WHIMS), reported a significantly increased risk of dementia, breast cancer, CVD, and stroke following MHT [6]. This risk was particularly evident with the use of oCEE and medroxyprogesterone acetate in women over the age of 65 [6]. As a result of these and other risks, many women stopped taking MHT.

Preclinical data reveal a complex relationship between estrogen and neuron health. Cell culture studies suggest that estrogen is beneficial in maintaining neuronal integrity [7]. However, estrogens may accelerate damage in mitochondria and calcium channels, which are thought to have a critical role in the development of cognitive impairment, a key characteristic of dementia. Older women may be more susceptible to this type of neuronal pathology, which might explain WHIMS findings. Estrogens can also have a negative effect on blood vessels, supporting their maintenance, but accelerating pathology once present [8]. CVD and dementia share risk factors; therefore, with a higher likelihood of pathology being present, based on WHIMS and several other RCTs, MHT may not be appropriate for older women (over age 65).

The Early versus Late Intervention Trial with Estradiol (ELITE) and KEEPS RCTs both showed no cognitive benefit or decline when MHT was initiated at the time of menopause (in KEEPS), early (within 6 years after menopause) or when it was initiated late (>10 years after menopause, in ELITE [9]). In these healthy women, later MHT conferred no negative effects on cognition. These RCTs also contradicted the “window of opportunity theory,” which states that MHT benefits brain function when administered close to the age of menopause [6,7].

In ELITE (which had also included healthy postmenopausal women without CVD or diabetes) after 5 years of oral estradiol, women did show a reduction in carotid intima thickness and LDL cholesterol levels (both risk factors for atherosclerosis and CVD) when they had been given MHT within 6 years of menopause, but not after MHT had been given >10 years after menopause [9]. In stratified later analyses of WHIMS (WHIMSY), risk for CVD was also reduced with MHT in women in a younger age group (50–55 years of age). Similar to KEEPS, in WHIMSY, however, also, no difference was found after MHT or placebo on cognitive tests 7 years after the trial had stopped when these women were, on average, 67 years of age [10,11].

Women who enroll in RCT or observational studies are not necessarily representative of the general population. To further investigate this association in the general population, meta-analysis was performed using data from National Registries where pharmacy data of prescribed MHT were linked with medical records of diagnosed dementia [11]. A slight increased risk of dementia was seen after 5–10 and 10+ years prescription of combination MHT (estrogen with a progestagen) in women younger than 60 years.

ESHRE guidelines suggested that for early or premature surgical menopausal women without a womb, MHT with estrogen alone benefits the brain [2]. However, RCTs for this group were small and/or of short duration, and observational data from this group might be confounded as these women often have a worse risk profile for dementia, with higher prevalence of smoking and obesity, and lower socioeconomic position (SEP), all risk factors for later life CVD and dementia [12]. In our registry meta-analyses for estrogen without progestogens, no overall increased benefits or risks of MHT were seen on dementia risk in women younger than 60 years [11]. Importantly, we could not analyze data for premature and early menopause and MHT separately from women undergoing menopause at a later age. Longer-term RCT with MHT for premature and early menopausal women are needed to further illuminate risk or benefit for dementia for this group [3].

Reassuringly, the data from RCT combined with longitudinal follow-up studies (KEEPS-Continuation and WHIMSY) suggest that MHT has a good brain safety profile later in life, up to 10 years after treatment cessation in midlife, when given close to the age at menopause for fewer than 5 years in healthy women. Women of different ethnicities and/or low SEP, who are at higher risk for diabetes and CVD and who may experience more severe vasomotor symptoms with an earlier age at (surgical) menopause, need to be studied more comprehensively, due to the lack of existing data, as highlighted by NICE [3]. While MHT can offer vasomotor symptom and sleep disturbance relief and protection against osteoporosis, clinicians must balance such benefits with some risks, including breast and endometrial cancer and, with oral MHT, for stroke and venous thrombotic events [3].

## Abbreviations

**:**
CVD: cardiovascular disease
ELITE: Early versus Late Intervention Trial with Estradiol
ESHRE: European Society of Human Reproduction and Embryology
KEEPS: Kronos Early Estrogen Prevention Study
MHT: menopausal hormone therapy
NICE: National Institute for Health and Care Excellence
oCEE: oral conjugated equine estrogens
RCTs: randomized controlled trials
SEP: socioeconomic position
WHI: Women’s Health Initiative Study
WHIMS: Women’s Health Initiative Memory Study
